# Addiction

**DOI:** 10.1192/j.eurpsy.2025.82

**Published:** 2025-08-26

**Authors:** J. G. Bramness

**Affiliations:** Group of Psychiatric Research, UiT The Arctic University of Norway, Tromsø, Norway

## Abstract

**Abstract:**

Bach et al (2024) investigated in a study published in Biological Psychiatry the effects of psychosocial stress on brain activation in individuals with alcohol use disorder (AUD), finding that stress heightened alcohol cue-induced activation in the left anterior insula, a region associated with salience attribution and goal-directed behavior. The activation correlated with alcohol craving and subsequent alcohol use, suggesting a neurobiological mechanism linking stress, cue reactivity, and relapse. Hassen et al (2024) performed a multi-center study examining cocaine use across Europe, revealing prevalence rates with a general upward trend, particularly among youth, marginalized groups, and opiate-dependent individuals. The study was published in European Addiction Research. Leonhardt et al (2024) investigated individuals with co-occurring substance use disorder and mental illness during the COVID-19 pandemic in Norway and found dramatically higher all-cause mortality rates, despite no increased risk of SARS-CoV-2 infection. This excess mortality highlights the vulnerability of this population and suggests that factors beyond the virus itself contributed to their elevated death rates. The study of Qeadan et al (2024) published in Addiction was one of many studies indicating that GIP/GLP-1 receptor agonist were effective against alcohol and other abuse. This study found lower rates of opioid overdose and alcohol intoxication. The findings support the use of these drugs at least in AUD.

**Disclosure of Interest:**

None Declared

